# An analogue of indapamide: crystal structure and Hirshfeld surface analysis of 3-chloro-4-(*N*,*N*-diethynylsulfamo­yl)-*N*-(2-meth­yl­indolin-1-yl)benzamide

**DOI:** 10.1107/S2056989026003580

**Published:** 2026-04-10

**Authors:** Youssef Ramli, Wedad Al Garadi, El Mokhtar Essassi, Camille Kalonji Mubengayi, Abdulsalam Alsubari, Joel T. Mague

**Affiliations:** a Laboratory of Medicinal Chemistry Drug Sciences Research Center Faculty of Medicine and Pharmacy Mohammed V University in Rabat Morocco; b Laboratoire de Chimie Organique Heterocyclique Faculté des Sciences, Université Mohammed V, Rabat, Morocco; c Laboratoire de Chimie et Biochimie, Institut Superieur des Techniques Medicales, Kinshasa, Republique Democratique, Congo; d Laboratory of Medicinal Chemistry, Faculty of Clinical Pharmacy, 21 September University, Yemen; e Department of Chemistry Tulane University New Orleans LA 70118 USA; Katholieke Universiteit Leuven, Belgium

**Keywords:** crystal structure, indole, benzamide, alkyne, sulfamo­yl, hydrogen bond, Hirshfeld surface

## Abstract

The title mol­ecule, C_22_H_20_ClN_3_O_3_S, adopts a shallow cup-shaped conformation with the chloro­benzamide portion as the bottom. A puckering analysis of the five-membered ring indicates an envelope conformation. In the crystal, helical chains along the *a*-axis direction are formed by N—H⋯O hydrogen bonds reinforced by C—H⋯π(ring) and weak π-stacking inter­actions. No directed inter­actions between chains appear to exist. A Hirshfeld surface analysis was performed.

## Abstract 2

The title mol­ecule adopts a shallow cup-shaped conformation with the chloro­benzamide portion as the bottom. In the crystal, helical chains along the *a*-axis direction are formed by N—H⋯O hydrogen bonds reinforced by C—H⋯π(ring) and weak π-stacking inter­actions. A Hirshfeld surface analysis was performed.

## 1. Chemical context

Indapamide, C_16_H_16_ClN_3_O_3_S, an indoline derivative, is a di­hydro­indole-based thia­zide-like diuretic used to treat hypertension and to manage heart failure. It is on the World Health Organization Model List of Essential Medicines. The mol­ecule contains a polar sulfamoyl chloro­benzamide moiety and a lipid-soluble methyl­indoline moiety. Chemically, indole derivatives demonstrating anti­viral activity are substituted at the 2-, 3-, 5-, and 6-positions of the nucleus. Moreover, various activities are associated with indole derivatives, including anti­viral (Kadam & Wilson, 2016 ▸). Some analogs have also been synthesized and evaluated for their industrial properties (*e.g*. Ettahiri *et al.*, 2024 ▸).

Drug discovery is a long and complicated process. The average cost of discovering a new medicine by traditional methods is $2.6 billion, and the complete workflow may take more than 12 years (Mohs & Greig, 2017 ▸). An alternative to new drug development is drug repositioning, using an existing drug for a new treatment that was not indicated before. It is of essential importance today to accelerate the drug discovery process and find solutions more quickly for the overburdened healthcare system and the increasing need for medicines. This practice has received immense attention during the COVID-19 pandemic.

**Figure 1:**
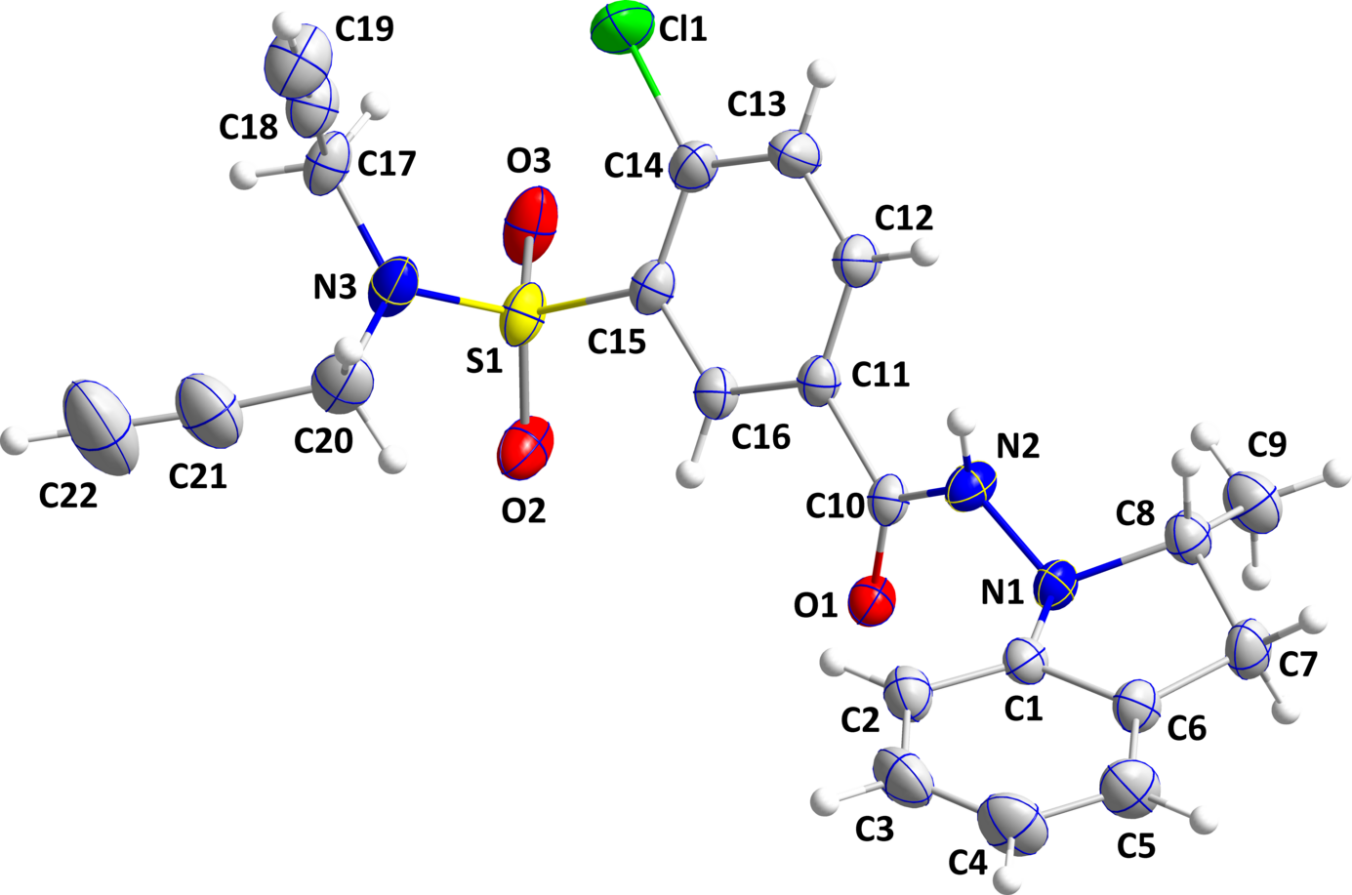
The title mol­ecule with labeling scheme and 30% probability ellipsoids.

As part of our work in this area including the use of indapamide analogues in a repositioning process (Ramli *et al.*, 2023 ▸; Al Garadi *et al.*, 2024 ▸), the title compound, C_22_H_20_ClN_3_O_3_S, was synthesized via an alkyl­ation reaction with propargyl bromide under phase-transfer catalysis conditions and its crystal structure is reported here (Fig. 1 ▸). A Hirshfeld surface analysis was performed to analyze the inter­molecular inter­actions.
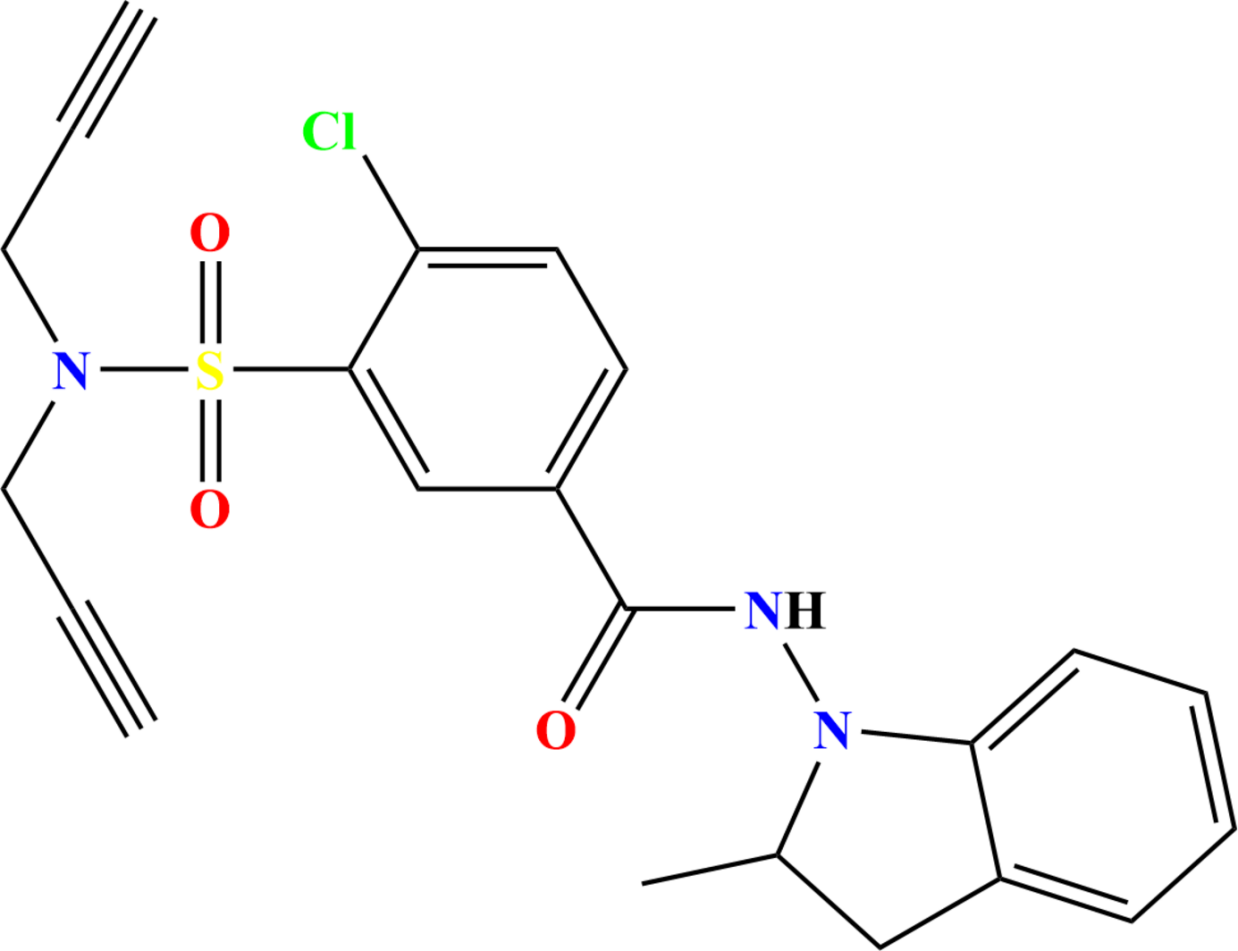


## 2. Structural commentary

The title mol­ecule adopts a shallow cup-shaped conformation with the 3-chloro­benzamide portion forming the bottom of the cup. A puckering analysis of the C1/C6/C7/C8/N1 ring (Cremer & Pople, 1975 ▸) yielded the parameters *Q*(2) = 0.317 (4) Å and φ(2) = 329.1 (8)° with the conformation characterized as an envelope on C8. The dihedral angle between the C1–C6 and C11–C16 rings is 63.2 (2)°. The propynyl groups point in opposite directions from the extension of the S1⋯N3 vector giving the N(CH_2_C≡CH)_2_ moiety a V-shape when viewed along the normal to the C15/S1/N3 plane (Fig. 1 ▸). All bond lengths and inter­bond angles appear as expected for the given formulation.

## 3. Supra­molecular features

**Figure 2:**
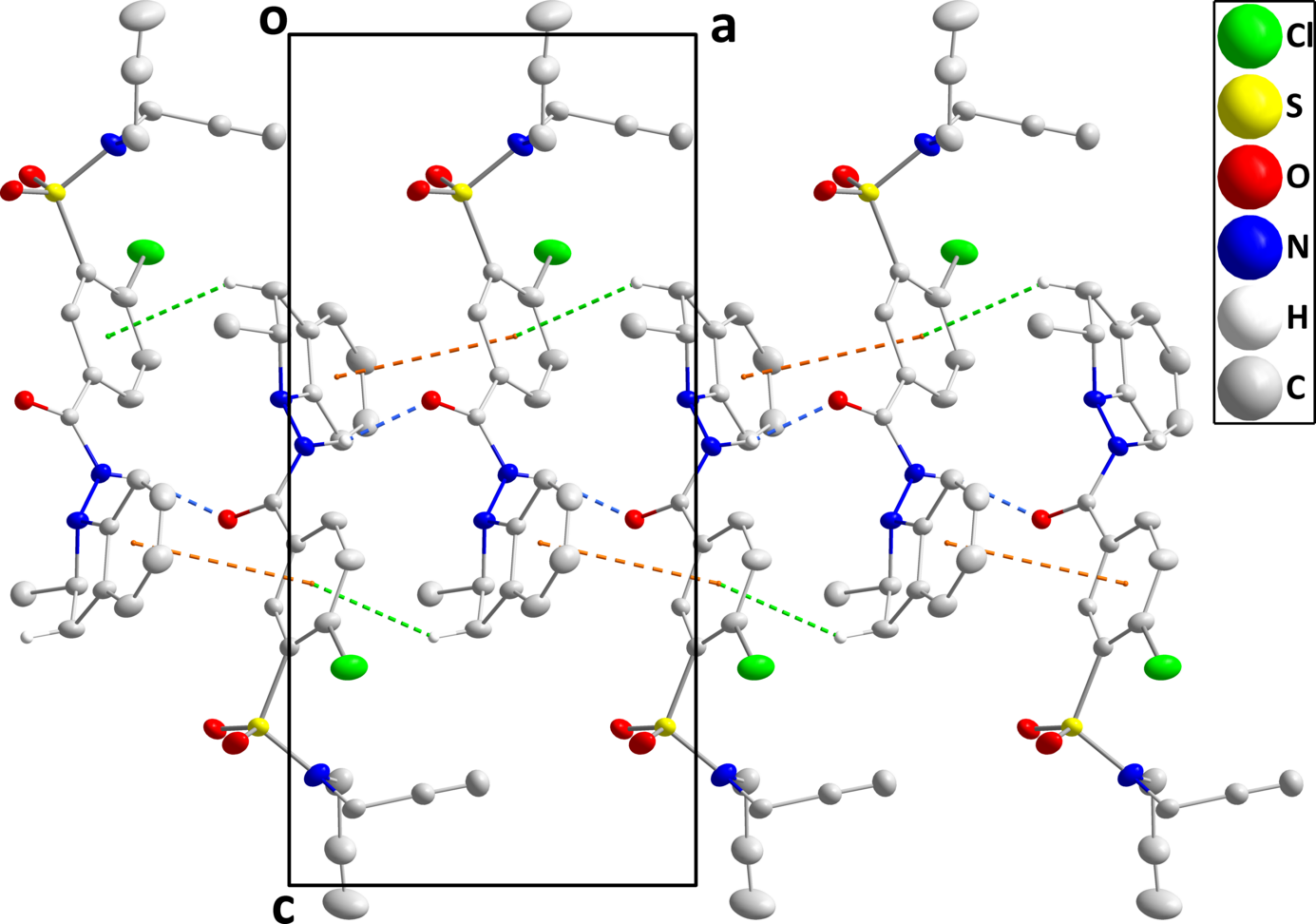
Detail of one chain viewed along the *b*-axis direction. N—H⋯O hydrogen bonds are depicted by blue dashed lines while π-stacking and C—H⋯π(ring) inter­actions are depicted, respectively, by orange and green dashed lines.

**Figure 3:**
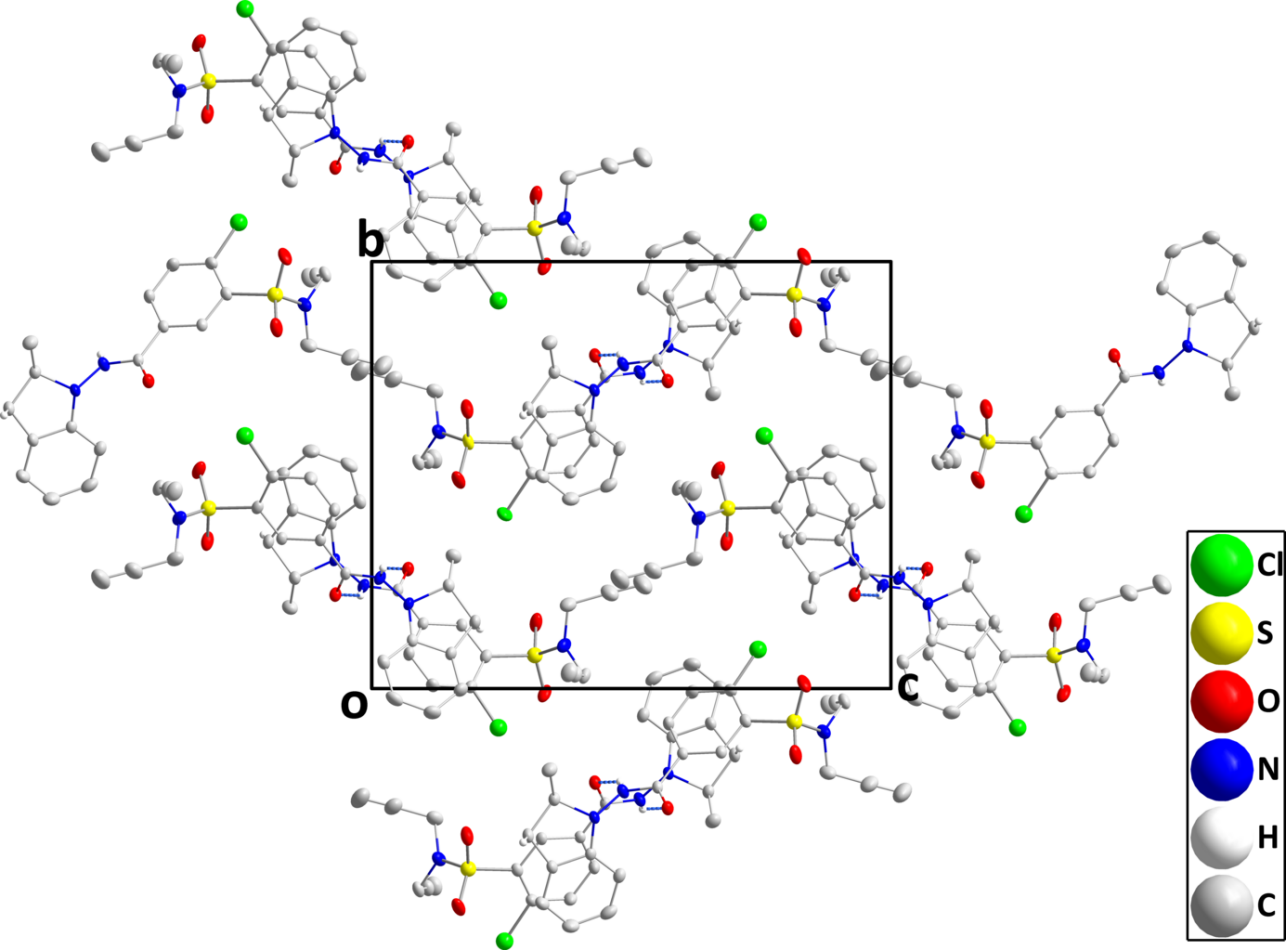
Packing viewed along the *a*-axis direction. Only the N—H⋯O hydrogen bonds (gray dashed lines) are shown for clarity.

In the crystal, helical chains extending along the *a*-axis direction are formed by N2—H2*A*⋯O1 hydrogen bonds (Table 1 ▸ and Fig. 2 ▸). These are strengthened by C7—H7*A*⋯*Cg*3 inter­actions (*Cg*3 is the centroid of the C11–C16 ring) and weak π-stacking inter­actions between the C1–C6 and C11–C16 rings [centroid⋯centroid = 3.859 (3) Å, dihedral angle = 7.2 (2)°, slippage = 0.959 Å] (Table 2 ▸ and Fig. 1 ▸). There appear to be no directed inter­actions between the chains (Fig. 3 ▸).

## 4. Database survey

**Figure 4:**
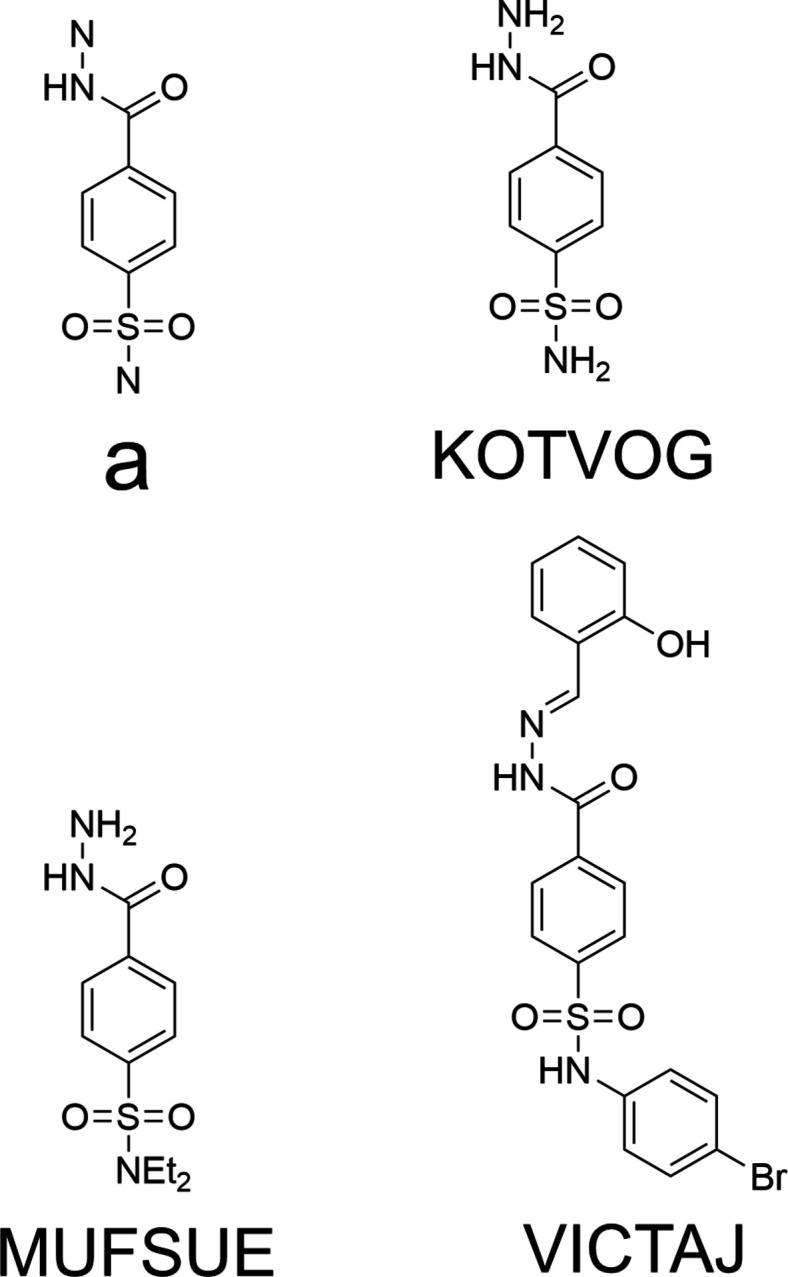
Search fragment (**a**) and structures of hits in the *Database Survey.*

A search of the Cambridge Structural Database (CSD, updated to Feb. 2026; Groom *et al.*, 2016 ▸) with the fragment shown in Fig. 4 ▸*a* yielded the three hits shown in Fig. 4 ▸. The cores of all three are essentially the same as that of the title mol­ecule, but the conformations of the full mol­ecule are different because of the different peripheral substituents and this leads to significantly different packings. Both KOTVOG (Poulsen & Healy, 2014 ▸) and MUFSUE (Gupta *et al.*, 2020 ▸) are considerably smaller than the title compound and are expected to display a more compact crystal packing. Furthermore, both have a terminal –NH_2_ group instead of the indolinyl moiety, and the former also has one on the sulfur atom, leading to greater opportunities for inter­molecular hydrogen bonding. In KOTVOG, therefore, inversion dimers are formed by N—H⋯O hydrogen bonds between the S—NH_2_ group of one mol­ecule and the carbonyl oxygen atom of the second. These units are connected by pairwise N—H⋯O hydrogen bonds between the same groups, forming chains of dimers that extend along the *c*-axis direction. The chains are connected by pairwise N—H⋯N hydrogen bonds between the hydrazinyl moieties in layers parallel to (1
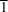
0), which contrasts with the packing in the title mol­ecule. There are also no π-stacking or C—H⋯π(ring) inter­actions. With only the hydrazinyl group present in MUFSUE, fewer hydrogen bonds can be formed. Pairwise N—H⋯O hydrogen bonds between the terminal NH_2_ and carbonyl groups form inversion dimers, which are connected by inversion-related N—H⋯N hydrogen bonds between the hydrazinyl moieties into ribbons extending along the *b*-axis direction. The ribbons are connected via weak C—H⋯O hydrogen bonds between one ethyl group and a sulfonyl oxygen atom. In YICTAJ (Liu *et al.*, 2023 ▸), the hydroxyl group makes an intra­molecular O—H⋯N hydrogen bond and is therefore not available for inter­molecular inter­actions, leaving only the two secondary amino groups for this latter purpose. One forms an N—H⋯O hydrogen bond with a dimethylformamide solvent mol­ecule, the other does not. The only inter­molecular inter­action appears to be a weak C—H⋯O hydrogen bond generating chains extending along the *b*-axis direction.

## 5. Hirshfeld surface analysis

**Figure 5:**
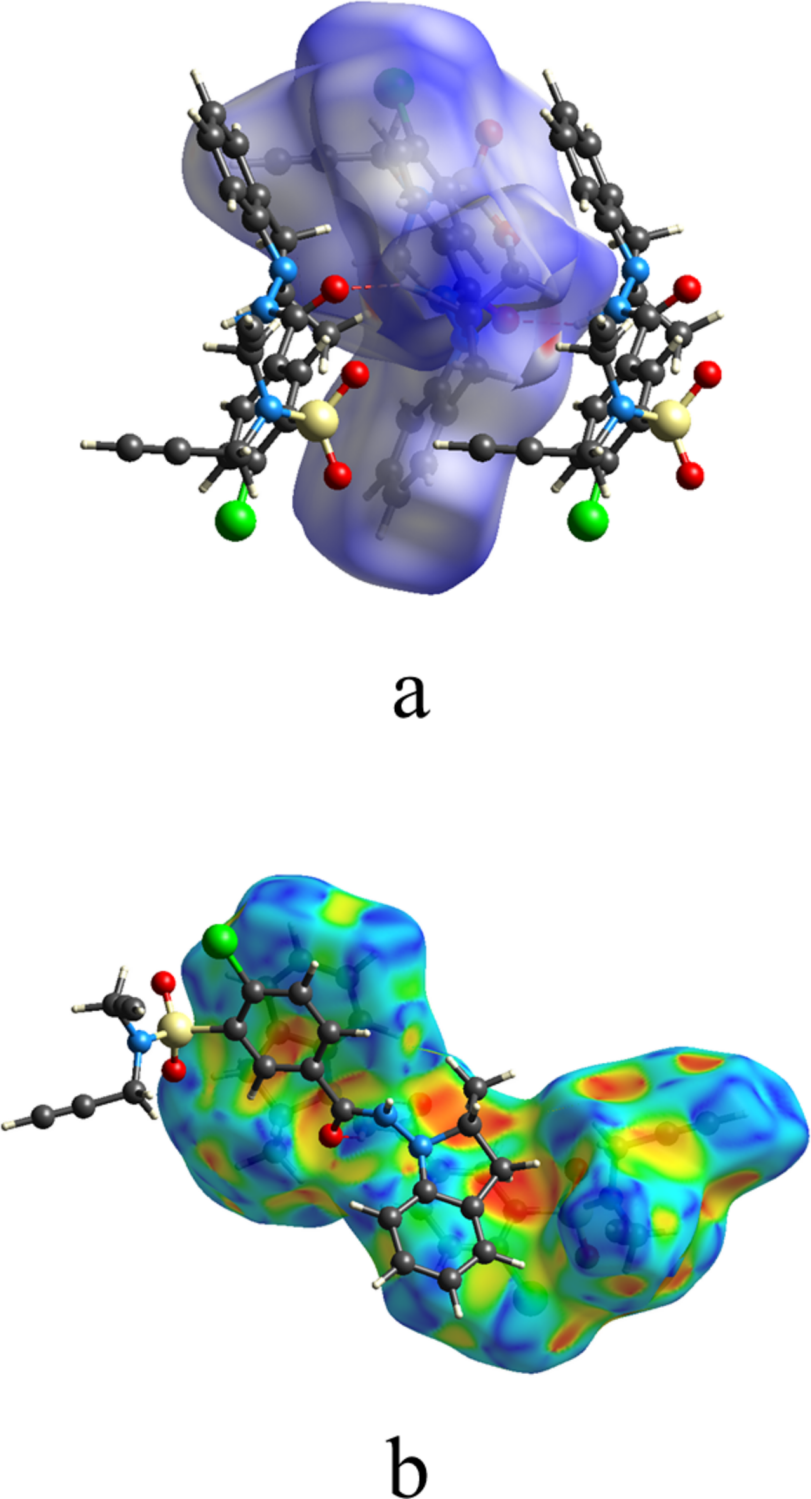
The *d*_norm_ (*a*) and shape surfaces (***b***) with neighboring mol­ecules. The N—H⋯O hydrogen bonds are depicted by red dashed lines.

**Figure 6:**
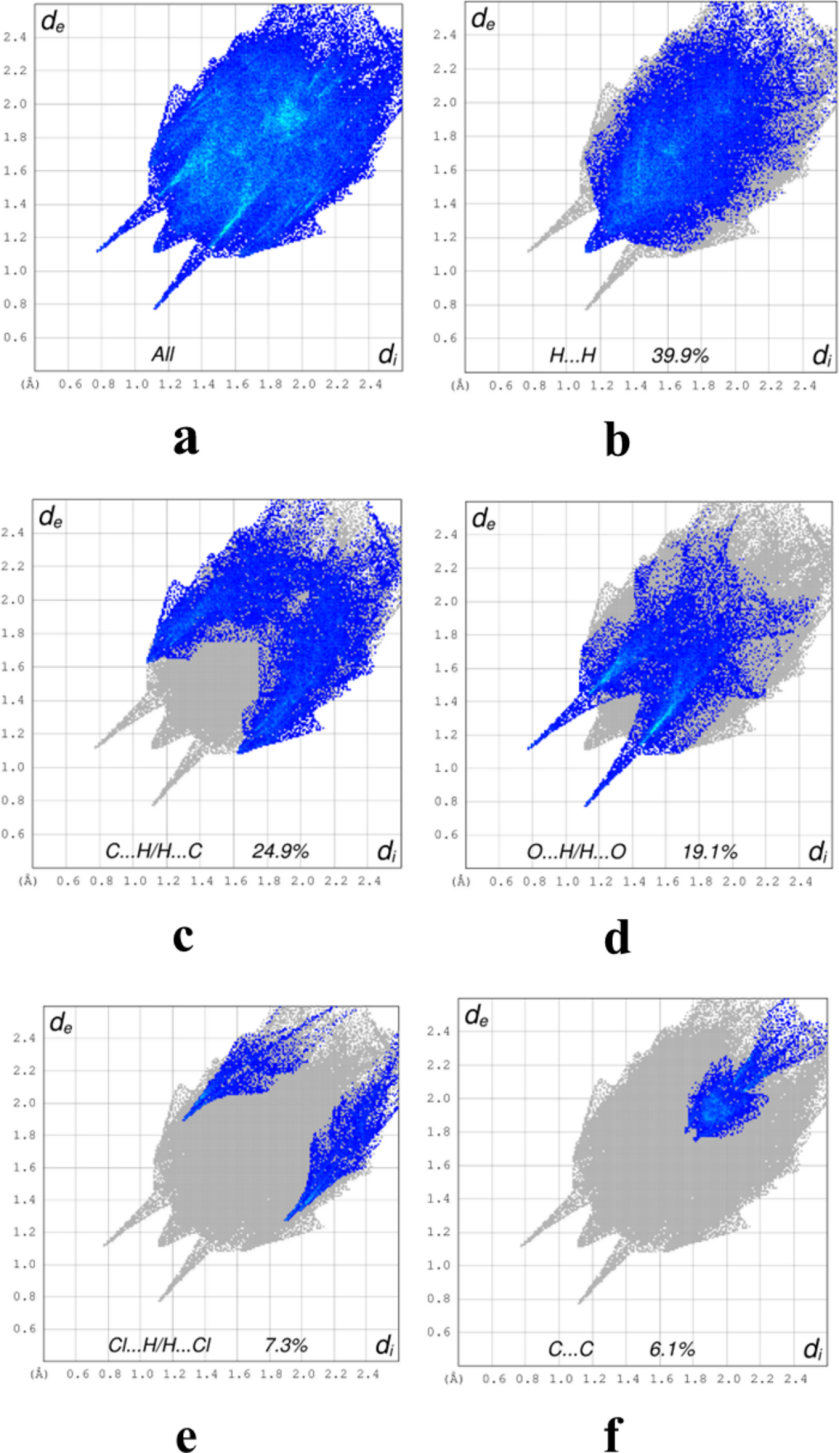
Two-dimensional fingerprint plots showing all inter­molecular inter­actions (*a*) and those resolved into H⋯H (*b*), C⋯H/H⋯C (*c*), O⋯H/H⋯O (*d*), Cl⋯H/H⋯Cl (*e*) and C⋯C (*f*) inter­actions.

To qu­antify the several inter­molecular inter­actions in the title compound, a Hirshfeld surface (HS) analysis was performed with *CrystalExplorer* (Spackman *et al.*, 2021 ▸). Descriptions of the plots obtained and their inter­pretations have been published (Tan *et al.*, 2019 ▸). Fig. 5 ▸ shows a portion of one helical chain with the HS for the central mol­ecule plotted over *d*_norm_ and over the shape function. The dark red spot in the former clearly indicates the N—H⋯O hydrogen bonds while the π-stacking inter­action shown in Fig. 2 ▸ can be seen near the top left of the latter. The C—H⋯π(ring) inter­action shown in Fig. 2 ▸ can be seen just below the dark orange spot at the right center of Fig. 5 ▸*b*. The two-dimensional fingerprint plots are presented in Fig. 6 ▸ where Fig. 6 ▸*a* shows all inter­actions and Fig. 6 ▸*b* the H⋯H contacts, which comprise 39.9% of the total. This is as expected since the periphery of the mol­ecule consists mainly of hydrogen atoms. At 24.9% of the total are the C⋯H/H⋯C inter­actions (Fig. 6 ▸*c*), which appear as a pair of rounded peaks at *d*_e_ + *d*_i_ ≃ 2.7 Å superimposed on more diffuse peaks. The former can be associated with the C—H⋯π(ring) inter­actions, since the H⋯C distances in these contacts cover a relatively narrow range, while the latter represent various van der Waals H⋯C contacts. The pair of sharp spikes at *d*_e_ + *d*_i_ ≃ 2.2 Å (6*d*) are assigned to O⋯H/H⋯O contacts (19.1%) which are mainly the N—H⋯O hydrogen bonds. Since no N—H⋯Cl or C—H⋯Cl hydrogen bonds are reported, it may seem surprising that H⋯Cl contacts (Fig. 6 ▸*e*) amount to 7.3% of the total. However, the sum of the van der Waals radii for these two atoms is 2.95 Å and *d*_e_ + *d*_i_ ≃ 3.2 Å for the H⋯Cl contacts, so this represents no significant attractive inter­actions. Finally, the C⋯C contacts (Fig. 6 ▸*f*) contribute 6.1% and can be attributed largely to the π-stacking inter­actions noted in Section 3. All other atom⋯atom contacts make significantly smaller contributions.

## 6. Synthesis and crystallization

Indapamide (0.5 g, 1.36 mmol) and potassium bicarbonate (0.37 g, 2.70 mmol) were dissolved in di­methyl­formamide (10 mL), to which was added dropwise propargyl bromide (2.90 mmol) along with a catalytic amount of BTBA (benzyl tributyl ammonium bromide). Under reflux, the reaction was stirred for 2 h at 355 K. When the starting reagents had reacted completely, distilled water (100 ml) was added. The product precipitated in solid form, was filtered, dried and recrystallized from ethanol solution to afford colorless blocks.

Yield = 67%, m.p. = 440–442 K. FT-IR (ATR, cm^−1^): 3375 (CH proparg­yl), 3060–3080, (CH aromatic), 1765 (C=O); ^1^H NMR (500MHz, DMSO-*d*_6_): ppm 0.917–0.929 (*d*, 3H, CH_3_, indo), 3.22 (*t*, 2H, CH proparg­yl), 4,22 (*s*, 4H, N—CH_2_), 7.03–7.42 (*m*, 10H, Ar—H), 9.78 (*s*, 1H, NH); ^13^C NMR: 28.01 (N—CH_2_); 74.40 (CH proparg­yl); 69.71 (C—2Ph); 74.40 (C_q_ proparg­yl); 127.25, 128.00, 128.58, 140.15 (C—Ar), 172.73 (C=O).

## 7. Refinement

Crystal data, data collection and structure refinement details are summarized in Table 2 ▸. H-atoms attached to carbon were placed in calculated positions (C—H = 0.95–1.00 Å) while that attached to nitro­gen was placed in a location derived from a difference map and its coordinates adjusted to give N—H = 0.91 Å. All were included as riding contributions with isotropic displacement parameters 1.2–1.5 times those of the attached atoms. The crystal studied was refined as a two-component inversion twin (domain ratio = 94:6)..

## Supplementary Material

Crystal structure: contains datablock(s) global, I. DOI: 10.1107/S2056989026003580/vm2328sup1.cif

Structure factors: contains datablock(s) I. DOI: 10.1107/S2056989026003580/vm2328Isup2.hkl

Supporting information file. DOI: 10.1107/S2056989026003580/vm2328Isup3.cml

CCDC reference: 2544250

Additional supporting information:  crystallographic information; 3D view; checkCIF report

## Figures and Tables

**Table 1:** Hydrogen-bond geometry (Å, °) *Cg*3 is the centroid of the C11–C16 benzene ring.

*D*—H⋯*A*	*D*—H	H⋯*A*	*D*⋯*A*	*D*—H⋯*A*
N2—H2*A*⋯O1^i^	0.91	1.98	2.812 (4)	152
C7—H7a⋯*Cg*3^i^	0.97	2.90	3.753 (5)	147

Symmetry code: (i) 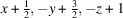.

**Table 2:** Experimental details

Crystal data	
Chemical formula	C_22_H_20_ClN_3_O_3_S
*M*_r_	441.92
Crystal system, space group	Orthorhombic, *P*2_1_2_1_2_1_
Temperature (K)	296
*a*, *b*, *c* (Å)	8.4412 (2), 14.5051 (3), 17.6477 (4)
*V* (Å^3^)	2160.79 (8)
*Z*	4
Radiation type	Cu *K*α
μ (mm^−1^)	2.71
Crystal size (mm)	0.27 × 0.12 × 0.07

Data collection	
Diffractometer	Bruker D8 VENTURE PHOTON 100 CMOS
Absorption correction	Multi-scan *SADABS* (Krause *et al.*, 2015 ▸)
*T*_min_, *T*_max_	0.75, 0.84
No. of measured, independent and observed [*I* > 2σ(*I*)] reflections	15363, 3788, 3223
*R*_int_	0.051
(sin θ/λ)_max_ (Å^−1^)	0.595

Refinement	
*R*[*F*^2^ > 2σ(*F*^2^)], *wR*(*F*^2^), *S*	0.041, 0.095, 1.07
No. of reflections	3788
No. of parameters	273
H-atom treatment	H-atom parameters constrained
Δρ_max_, Δρ_min_ (e Å^−3^)	0.21, −0.24
Absolute structure	Refined as an inversion twin
Absolute structure parameter	0.06 (3)

Computer programs: *APEX3* and *SAINT* (Bruker, 2016 ▸), *SAINT* (Bruker, 2016 ▸), *SHELXT/5* (Sheldrick, 2015*a* ▸), *SHELXL 2018/3* (Sheldrick, 2015*b* ▸), *DIAMOND* (Brandenburg & Putz, 2012 ▸) and *SHELXTL* (Sheldrick, 2008 ▸).

## supplementary crystallographic information

### 3-Chloro-4-(N,N-diethynylsulfamoyl)-N-(2-methylindolin-1-yl)benzamide Crystal data

**Table:**

C_22_H_20_ClN_3_O_3_S	*D*_x_ = 1.358 Mg m^−^^3^
*M**_r_* = 441.92	Cu *K*α radiation, λ = 1.54178 Å
Orthorhombic, *P*2_1_2_1_2_1_	Cell parameters from 9996 reflections
*a* = 8.4412 (2) Å	θ = 4.0–66.6°
*b* = 14.5051 (3) Å	µ = 2.71 mm^−^^1^
*c* = 17.6477 (4) Å	*T* = 296 K
*V* = 2160.79 (8) Å^3^	Plate, colourless
*Z* = 4	0.27 × 0.12 × 0.07 mm
*F*(000) = 920	

### 3-Chloro-4-(N,N-diethynylsulfamoyl)-N-(2-methylindolin-1-yl)benzamide Data collection

**Table:**

Bruker D8 VENTURE PHOTON 100 CMOS diffractometer	3788 independent reflections
Radiation source: INCOATEC IµS micro–focus source	3223 reflections with *I* > 2σ(*I*)
Mirror monochromator	*R*_int_ = 0.051
Detector resolution: 10.4167 pixels mm^-1^	θ_max_ = 66.7°, θ_min_ = 4.0°
ω scans	*h* = −10→10
Absorption correction: multi-scan *SADABS* (Krause *et al.*, 2015)	*k* = −17→17
*T*_min_ = 0.75, *T*_max_ = 0.84	*l* = −21→20
15363 measured reflections	

### 3-Chloro-4-(N,N-diethynylsulfamoyl)-N-(2-methylindolin-1-yl)benzamide Refinement

**Table:**

Refinement on *F*^2^	Secondary atom site location: difference Fourier map
Least-squares matrix: full	Hydrogen site location: mixed
*R*[*F*^2^ > 2σ(*F*^2^)] = 0.041	H-atom parameters constrained
*wR*(*F*^2^) = 0.095	*w* = 1/[σ^2^(*F*_o_^2^) + (0.0338*P*)^2^ + 0.5813*P*] where *P* = (*F*_o_^2^ + 2*F*_c_^2^)/3
*S* = 1.07	(Δ/σ)_max_ < 0.001
3788 reflections	Δρ_max_ = 0.21 e Å^−^^3^
273 parameters	Δρ_min_ = −0.23 e Å^−^^3^
0 restraints	Absolute structure: Refined as an inversion twin
Primary atom site location: dual	Absolute structure parameter: 0.06 (3)

### 3-Chloro-4-(N,N-diethynylsulfamoyl)-N-(2-methylindolin-1-yl)benzamide Special details

**Table:**

Geometry. All esds (except the esd in the dihedral angle between two l.s. planes) are estimated using the full covariance matrix. The cell esds are taken into account individually in the estimation of esds in distances, angles and torsion angles; correlations between esds in cell parameters are only used when they are defined by crystal symmetry. An approximate (isotropic) treatment of cell esds is used for estimating esds involving l.s. planes.
Refinement. Refinement of F^2^ against ALL reflections. The weighted R-factor wR and goodness of fit S are based on F^2^, conventional R-factors R are based on F, with F set to zero for negative F^2^. The threshold expression of F^2^ > 2sigma(F^2^) is used only for calculating R-factors(gt) etc. and is not relevant to the choice of reflections for refinement. R-factors based on F^2^ are statistically about twice as large as those based on F, and R- factors based on ALL data will be even larger. H-atoms attached to carbon were placed in calculated positions (C—H = 0.95 - 1.00 Å) while that attached to nitrogen was placed in a location derived from a difference map and its coordinates adjusted to give N—H = 0.91 Å. All were included as riding contributions with isotropic displacement parameters 1.2 - 1.5 times those of the attached atoms. Refined as a 2-component inversion twin.

### 3-Chloro-4-(N,N-diethynylsulfamoyl)-N-(2-methylindolin-1-yl)benzamide Fractional atomic coordinates and isotropic or equivalent isotropic displacement parameters (Å^2^)

**Table:**

	*x*	*y*	*z*	*U*_iso_*/*U*_eq_	
Cl1	0.6475 (2)	0.40841 (8)	0.25598 (7)	0.0937 (5)	
S1	0.42430 (12)	0.57786 (8)	0.18596 (5)	0.0551 (3)	
O1	0.3490 (3)	0.77986 (19)	0.43008 (14)	0.0479 (6)	
O2	0.3177 (4)	0.6547 (2)	0.18362 (17)	0.0717 (9)	
O3	0.3681 (4)	0.4885 (2)	0.16709 (17)	0.0746 (10)	
N1	0.4803 (4)	0.8015 (2)	0.57124 (16)	0.0456 (7)	
N2	0.5376 (4)	0.7404 (2)	0.51591 (16)	0.0490 (8)	
H2A	0.637517	0.717908	0.521840	0.059*	
N3	0.5683 (5)	0.6011 (2)	0.1296 (2)	0.0639 (10)	
C1	0.5577 (5)	0.8871 (3)	0.57830 (19)	0.0431 (8)	
C2	0.6292 (5)	0.9380 (3)	0.5230 (2)	0.0568 (11)	
H2	0.637507	0.916316	0.473555	0.068*	
C3	0.6892 (6)	1.0237 (4)	0.5436 (3)	0.0758 (15)	
H3	0.738360	1.060132	0.507165	0.091*	
C4	0.6776 (7)	1.0558 (3)	0.6168 (3)	0.0825 (16)	
H4	0.718053	1.113514	0.629203	0.099*	
C5	0.6061 (6)	1.0027 (3)	0.6716 (3)	0.0702 (13)	
H5	0.598392	1.024133	0.721099	0.084*	
C6	0.5462 (5)	0.9178 (3)	0.65258 (19)	0.0508 (9)	
C7	0.4650 (6)	0.8443 (3)	0.6988 (2)	0.0600 (11)	
H7A	0.355168	0.860240	0.708395	0.072*	
H7B	0.518555	0.834821	0.746806	0.072*	
C8	0.4776 (5)	0.7588 (3)	0.6483 (2)	0.0507 (9)	
H8	0.579410	0.728395	0.657652	0.061*	
C9	0.3472 (6)	0.6902 (3)	0.6575 (3)	0.0700 (13)	
H9A	0.247022	0.719966	0.649403	0.105*	
H9B	0.350362	0.665124	0.707849	0.105*	
H9C	0.360279	0.641427	0.621309	0.105*	
C10	0.4604 (4)	0.7306 (3)	0.44967 (18)	0.0397 (8)	
C11	0.5168 (4)	0.6523 (3)	0.40122 (19)	0.0414 (8)	
C12	0.6103 (5)	0.5817 (3)	0.4283 (2)	0.0569 (10)	
H12	0.647610	0.583840	0.477844	0.068*	
C13	0.6493 (6)	0.5077 (3)	0.3826 (3)	0.0679 (13)	
H13	0.712371	0.460407	0.401498	0.082*	
C14	0.5944 (6)	0.5042 (3)	0.3088 (2)	0.0578 (10)	
C15	0.5012 (4)	0.5754 (3)	0.2801 (2)	0.0469 (9)	
C16	0.4624 (4)	0.6486 (3)	0.32689 (19)	0.0419 (8)	
H16	0.399143	0.696003	0.308369	0.050*	
C17	0.6593 (5)	0.5297 (3)	0.0899 (2)	0.0589 (11)	
H17A	0.644749	0.537089	0.035748	0.071*	
H17B	0.618394	0.469623	0.103986	0.071*	
C18	0.8292 (6)	0.5333 (4)	0.1074 (2)	0.0639 (12)	
C19	0.9632 (7)	0.5378 (4)	0.1199 (3)	0.0846 (16)	
H19	1.071132	0.541463	0.130035	0.101*	
C20	0.6230 (7)	0.6965 (3)	0.1216 (3)	0.0764 (14)	
H20A	0.728463	0.702277	0.142864	0.092*	
H20B	0.552950	0.737214	0.149446	0.092*	
C21	0.6260 (9)	0.7239 (4)	0.0417 (4)	0.0961 (19)	
C22	0.6390 (13)	0.7438 (6)	−0.0220 (5)	0.151 (4)	
H22	0.649338	0.759671	−0.072812	0.181*	

### 3-Chloro-4-(N,N-diethynylsulfamoyl)-N-(2-methylindolin-1-yl)benzamide Atomic displacement parameters (Å^2^)

**Table:**

	*U*^11^	*U*^22^	*U*^33^	*U*^12^	*U*^13^	*U*^23^
Cl1	0.1511 (14)	0.0585 (7)	0.0715 (7)	0.0233 (8)	0.0086 (8)	−0.0165 (6)
S1	0.0507 (5)	0.0763 (7)	0.0383 (4)	−0.0105 (6)	0.0011 (4)	−0.0140 (5)
O1	0.0415 (14)	0.0608 (16)	0.0416 (13)	0.0051 (13)	−0.0027 (12)	−0.0029 (12)
O2	0.0584 (18)	0.110 (2)	0.0466 (15)	0.0233 (18)	−0.0116 (15)	−0.0128 (18)
O3	0.078 (2)	0.090 (2)	0.0564 (18)	−0.0401 (19)	0.0098 (15)	−0.0251 (16)
N1	0.0547 (19)	0.0486 (17)	0.0336 (13)	−0.0008 (15)	−0.0017 (13)	−0.0081 (13)
N2	0.0456 (18)	0.0597 (19)	0.0417 (15)	0.0078 (16)	−0.0079 (14)	−0.0137 (14)
N3	0.072 (2)	0.061 (2)	0.0580 (19)	−0.013 (2)	0.0223 (19)	−0.0129 (17)
C1	0.044 (2)	0.049 (2)	0.0365 (17)	0.0042 (17)	−0.0064 (16)	0.0009 (15)
C2	0.059 (2)	0.065 (3)	0.047 (2)	0.001 (2)	−0.0013 (18)	0.0086 (19)
C3	0.078 (3)	0.064 (3)	0.085 (4)	−0.007 (3)	0.003 (3)	0.029 (3)
C4	0.103 (4)	0.054 (3)	0.091 (4)	−0.018 (3)	−0.013 (3)	0.000 (3)
C5	0.095 (4)	0.056 (2)	0.060 (3)	−0.009 (3)	−0.014 (3)	−0.011 (2)
C6	0.062 (2)	0.053 (2)	0.0380 (16)	0.000 (2)	−0.0078 (17)	−0.0053 (17)
C7	0.080 (3)	0.063 (3)	0.0374 (19)	−0.005 (2)	0.001 (2)	−0.0046 (18)
C8	0.056 (2)	0.055 (2)	0.0408 (18)	−0.001 (2)	−0.0037 (17)	0.0015 (17)
C9	0.075 (3)	0.063 (3)	0.072 (3)	−0.012 (3)	−0.004 (3)	0.009 (2)
C10	0.0371 (19)	0.048 (2)	0.0335 (16)	−0.0052 (17)	−0.0003 (14)	−0.0017 (15)
C11	0.043 (2)	0.045 (2)	0.0361 (17)	−0.0052 (18)	0.0005 (15)	−0.0025 (15)
C12	0.076 (3)	0.058 (2)	0.0371 (17)	0.011 (2)	−0.0050 (18)	−0.0024 (19)
C13	0.097 (4)	0.055 (3)	0.052 (2)	0.024 (3)	−0.002 (3)	0.004 (2)
C14	0.078 (3)	0.048 (2)	0.048 (2)	0.000 (2)	0.011 (2)	−0.0049 (18)
C15	0.044 (2)	0.054 (2)	0.0421 (17)	−0.006 (2)	0.0031 (15)	−0.0076 (18)
C16	0.0395 (19)	0.049 (2)	0.0372 (18)	−0.0048 (16)	−0.0032 (15)	−0.0011 (15)
C17	0.062 (3)	0.072 (3)	0.043 (2)	−0.010 (2)	0.0067 (19)	−0.017 (2)
C18	0.062 (3)	0.078 (3)	0.052 (2)	−0.009 (3)	−0.002 (2)	−0.008 (2)
C19	0.064 (3)	0.109 (4)	0.080 (3)	−0.009 (3)	−0.005 (3)	−0.014 (3)
C20	0.085 (4)	0.062 (3)	0.083 (3)	−0.017 (3)	0.015 (3)	−0.010 (3)
C21	0.112 (5)	0.081 (4)	0.095 (4)	−0.022 (4)	−0.008 (4)	0.020 (3)
C22	0.230 (11)	0.117 (6)	0.106 (6)	−0.047 (7)	−0.025 (6)	0.036 (5)

### 3-Chloro-4-(N,N-diethynylsulfamoyl)-N-(2-methylindolin-1-yl)benzamide Geometric parameters (Å, º)

**Table:**

Cl1—C14	1.731 (4)	C7—H7B	0.9700
S1—O3	1.420 (3)	C8—C9	1.493 (6)
S1—O2	1.433 (3)	C8—H8	0.9800
S1—N3	1.607 (4)	C9—H9A	0.9600
S1—C15	1.783 (4)	C9—H9B	0.9600
O1—C10	1.230 (4)	C9—H9C	0.9600
N1—N2	1.405 (4)	C10—C11	1.499 (5)
N1—C1	1.408 (5)	C11—C12	1.379 (5)
N1—C8	1.494 (5)	C11—C16	1.391 (5)
N2—C10	1.346 (4)	C12—C13	1.381 (6)
N2—H2A	0.9100	C12—H12	0.9300
N3—C20	1.465 (6)	C13—C14	1.385 (6)
N3—C17	1.467 (5)	C13—H13	0.9300
C1—C2	1.365 (5)	C14—C15	1.395 (6)
C1—C6	1.388 (5)	C15—C16	1.384 (5)
C2—C3	1.391 (7)	C16—H16	0.9300
C2—H2	0.9300	C17—C18	1.468 (6)
C3—C4	1.376 (8)	C17—H17A	0.9700
C3—H3	0.9300	C17—H17B	0.9700
C4—C5	1.376 (7)	C18—C19	1.154 (7)
C4—H4	0.9300	C19—H19	0.9300
C5—C6	1.373 (6)	C20—C21	1.465 (8)
C5—H5	0.9300	C20—H20A	0.9700
C6—C7	1.507 (6)	C20—H20B	0.9700
C7—C8	1.531 (5)	C21—C22	1.165 (9)
C7—H7A	0.9700	C22—H22	0.9300
			
O3—S1—O2	119.5 (2)	C7—C8—H8	109.1
O3—S1—N3	107.43 (18)	C8—C9—H9A	109.5
O2—S1—N3	107.1 (2)	C8—C9—H9B	109.5
O3—S1—C15	108.8 (2)	H9A—C9—H9B	109.5
O2—S1—C15	105.70 (18)	C8—C9—H9C	109.5
N3—S1—C15	107.79 (19)	H9A—C9—H9C	109.5
N2—N1—C1	117.2 (3)	H9B—C9—H9C	109.5
N2—N1—C8	112.1 (3)	O1—C10—N2	123.6 (3)
C1—N1—C8	107.0 (3)	O1—C10—C11	121.5 (3)
C10—N2—N1	120.2 (3)	N2—C10—C11	114.9 (3)
C10—N2—H2A	120.7	C12—C11—C16	119.1 (3)
N1—N2—H2A	117.7	C12—C11—C10	123.2 (3)
C20—N3—C17	117.1 (4)	C16—C11—C10	117.5 (3)
C20—N3—S1	119.8 (3)	C11—C12—C13	120.8 (4)
C17—N3—S1	122.9 (3)	C11—C12—H12	119.6
C2—C1—C6	122.2 (4)	C13—C12—H12	119.6
C2—C1—N1	128.3 (3)	C12—C13—C14	119.9 (4)
C6—C1—N1	109.5 (3)	C12—C13—H13	120.1
C1—C2—C3	117.2 (4)	C14—C13—H13	120.1
C1—C2—H2	121.4	C13—C14—C15	120.2 (4)
C3—C2—H2	121.4	C13—C14—Cl1	116.7 (3)
C4—C3—C2	121.5 (5)	C15—C14—Cl1	123.0 (3)
C4—C3—H3	119.3	C16—C15—C14	119.0 (3)
C2—C3—H3	119.3	C16—C15—S1	117.1 (3)
C5—C4—C3	120.1 (4)	C14—C15—S1	123.9 (3)
C5—C4—H4	120.0	C15—C16—C11	121.0 (4)
C3—C4—H4	120.0	C15—C16—H16	119.5
C6—C5—C4	119.5 (4)	C11—C16—H16	119.5
C6—C5—H5	120.2	N3—C17—C18	112.7 (4)
C4—C5—H5	120.2	N3—C17—H17A	109.0
C5—C6—C1	119.5 (4)	C18—C17—H17A	109.0
C5—C6—C7	132.0 (4)	N3—C17—H17B	109.0
C1—C6—C7	108.4 (3)	C18—C17—H17B	109.0
C6—C7—C8	103.0 (3)	H17A—C17—H17B	107.8
C6—C7—H7A	111.2	C19—C18—C17	178.4 (6)
C8—C7—H7A	111.2	C18—C19—H19	180.0
C6—C7—H7B	111.2	N3—C20—C21	110.8 (4)
C8—C7—H7B	111.2	N3—C20—H20A	109.5
H7A—C7—H7B	109.1	C21—C20—H20A	109.5
C9—C8—N1	112.8 (3)	N3—C20—H20B	109.5
C9—C8—C7	115.1 (4)	C21—C20—H20B	109.5
N1—C8—C7	101.3 (3)	H20A—C20—H20B	108.1
C9—C8—H8	109.1	C22—C21—C20	175.4 (9)
N1—C8—H8	109.1	C21—C22—H22	180.0
			
C1—N1—N2—C10	−102.3 (4)	C6—C7—C8—N1	−28.9 (4)
C8—N1—N2—C10	133.4 (4)	N1—N2—C10—O1	8.2 (6)
O3—S1—N3—C20	163.9 (4)	N1—N2—C10—C11	−170.0 (3)
O2—S1—N3—C20	34.4 (4)	O1—C10—C11—C12	−162.4 (4)
C15—S1—N3—C20	−79.0 (4)	N2—C10—C11—C12	15.8 (5)
O3—S1—N3—C17	−21.8 (4)	O1—C10—C11—C16	13.2 (5)
O2—S1—N3—C17	−151.4 (3)	N2—C10—C11—C16	−168.6 (3)
C15—S1—N3—C17	95.3 (4)	C16—C11—C12—C13	−0.4 (6)
N2—N1—C1—C2	32.1 (6)	C10—C11—C12—C13	175.2 (4)
C8—N1—C1—C2	159.0 (4)	C11—C12—C13—C14	0.1 (8)
N2—N1—C1—C6	−150.6 (3)	C12—C13—C14—C15	0.6 (7)
C8—N1—C1—C6	−23.8 (4)	C12—C13—C14—Cl1	−179.4 (4)
C6—C1—C2—C3	−0.9 (6)	C13—C14—C15—C16	−1.1 (6)
N1—C1—C2—C3	176.0 (4)	Cl1—C14—C15—C16	178.9 (3)
C1—C2—C3—C4	0.2 (7)	C13—C14—C15—S1	−180.0 (4)
C2—C3—C4—C5	0.4 (9)	Cl1—C14—C15—S1	0.0 (5)
C3—C4—C5—C6	−0.2 (8)	O3—S1—C15—C16	−136.8 (3)
C4—C5—C6—C1	−0.4 (7)	O2—S1—C15—C16	−7.2 (3)
C4—C5—C6—C7	179.0 (5)	N3—S1—C15—C16	107.0 (3)
C2—C1—C6—C5	1.0 (7)	O3—S1—C15—C14	42.1 (4)
N1—C1—C6—C5	−176.4 (4)	O2—S1—C15—C14	171.7 (3)
C2—C1—C6—C7	−178.6 (4)	N3—S1—C15—C14	−74.1 (4)
N1—C1—C6—C7	4.0 (5)	C14—C15—C16—C11	0.8 (6)
C5—C6—C7—C8	−163.0 (5)	S1—C15—C16—C11	179.8 (3)
C1—C6—C7—C8	16.5 (5)	C12—C11—C16—C15	−0.1 (6)
N2—N1—C8—C9	−74.0 (4)	C10—C11—C16—C15	−175.9 (3)
C1—N1—C8—C9	156.2 (4)	C20—N3—C17—C18	52.7 (6)
N2—N1—C8—C7	162.5 (3)	S1—N3—C17—C18	−121.7 (4)
C1—N1—C8—C7	32.7 (4)	C17—N3—C20—C21	58.3 (6)
C6—C7—C8—C9	−150.9 (4)	S1—N3—C20—C21	−127.1 (5)

### 3-Chloro-4-(N,N-diethynylsulfamoyl)-N-(2-methylindolin-1-yl)benzamide Hydrogen-bond geometry (Å, º)

*Cg*3 is the centroid of the C11–C16 benzene ring.

**Table:**

*D*—H···*A*	*D*—H	H···*A*	*D*···*A*	*D*—H···*A*
N2—H2*A*···O1^i^	0.91	1.98	2.812 (4)	152
C7—H7a···*Cg*3^i^	0.97	2.90	3.753 (5)	147

Symmetry code: (i) *x*+1/2, −*y*+3/2, −*z*+1.
